# Acute Ischemic Stroke Secondary to Iron Deficiency Anemia: A Case Report

**DOI:** 10.1155/2012/487080

**Published:** 2012-05-09

**Authors:** Preema J. Mehta, Sherita Chapman, Annapurni Jayam-Trouth, Mohankumar Kurukumbi

**Affiliations:** ^1^Department of Neurology, Howard University Hospital, 2041 Georgia Ave., Washington, DC 20060, USA; ^2^Department of Neurology, Howard University College of Medicine, Washington, DC 20059, USA

## Abstract

A rare case of acute ischemic stroke in a young patient with iron deficiency anemia (IDA) is reported. IDA has been suggested to have an association with stroke, but few cases have proven it thus far. Three physiological mechanisms explaining IDA to ischemic stroke include a hypercoagulable state secondary to IDA, thrombocytosis secondary to IDA, and anemic hypoxia induced by IDA. Our paper shows an example of a hypoxia-induced stroke secondary to IDA in a young woman with menorrhagia. Thrombus formation was ruled out as the Magnetic Resonance Angiogram (MRA) showed no evidence. As all other known causes for stroke were ruled out, the patient's IDA is a reasonable cause for her stroke. Iron deficiency decreases the amount of hemoglobin, which consequently decreases the amount of oxygen in the blood resulting in low-oxygen delivery to the brain. This causes hypoxic conditions in the brain, leading to death of brain tissue. Thus, we suggest a possible relationship between IDA and ischemic stroke in young adults. Considering IDA as one of the risk factors for ischemic stroke and treating with timely transfusions would be an important step one can take to prevent stroke.

## 1. Introduction

Strokes are often considered a consequence of hypertension and atherosclerosis. Some rare causes of stroke also include systemic hypoperfusion, sickle cell anemia, cerebral venous sinus thrombosis, arterial fibrillation, and cocaine abuse. Iron deficiency anemia as a risk factor for acute ischemic stroke has been reported in only a few case reports [1]. The majority of these few cases show thrombosis secondary to IDA causing ischemic stroke [1], but in our case it was hypoxia-induced stroke secondary to IDA.

## 2. Case Presentation

A forty-seven-year-old female presented to the emergency department with complaints of weakness and numbness of two-hour duration in the left upper extremity as well as slurred speech. Her past medical history was remarkable for iron deficiency anemia and menorrhagia due to a uterine fibroid. She also had a history of chronic pulmonary heart disease and asthma. She denied taking any medications for these conditions and was also not taking any oral contraceptives. Her family history was negative for any neurological or hematological conditions.

**Figure 1:**
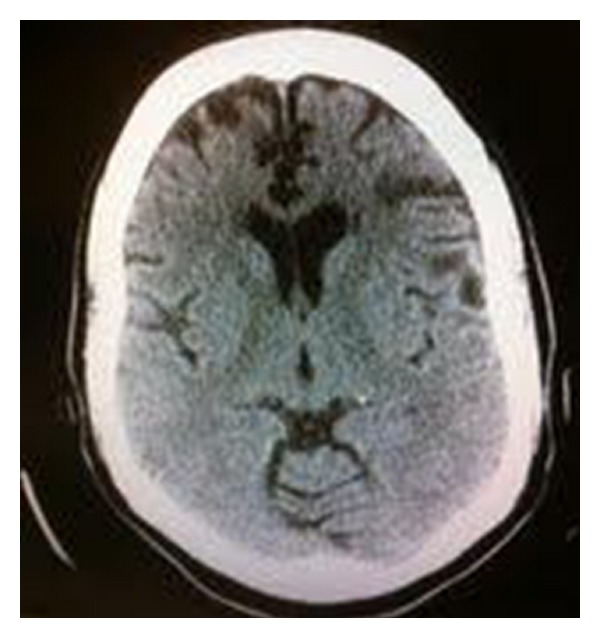
CT scan of the head without contrast showing old bifrontal encephalomalacia (left greater than right).

**Figure 2:**
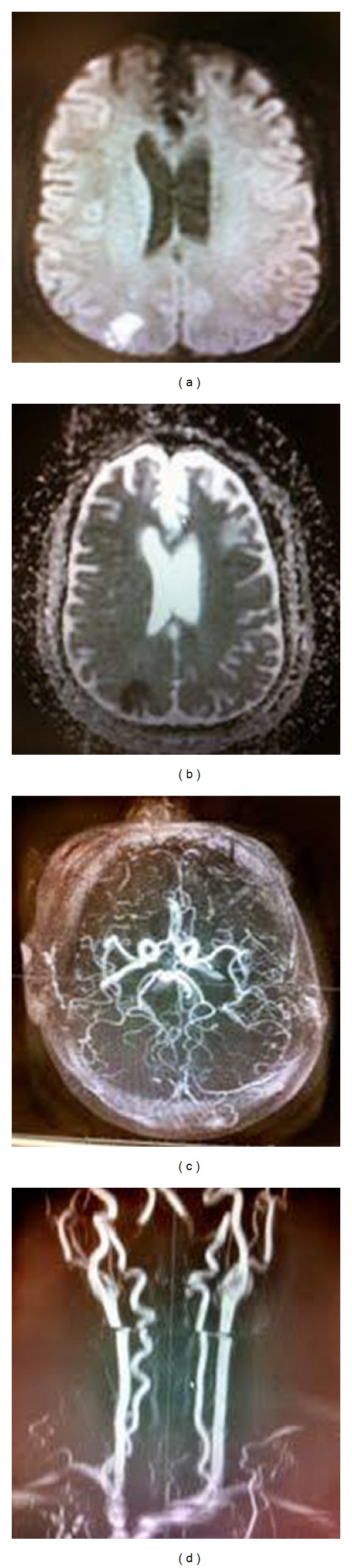
MRI Brain (a) DWI showing hyperintense lesion in the right MCA territory. (b) ADC showing mismatch in the same region confirming acute infarction. (c) and (d) MRA showing no evidence of thrombus formation or carotid dissection.

The clinical examination in the emergency room included mild dysarthria and decreased repetitive finger movements in her left upper extremity. The rest of the detailed neurological examination was normal. The patient was initiated with stroke protocol and CT of the head was done, which revealed an old bifrontal encephalomalacia with left greater than right, possibly secondary to previous trauma (Figure 1). MRI of the brain showed acute infarction in right middle cerebral artery (MCA) distribution (Figures 2(a) and 2(b)). MRA revealed no evidence of thrombus formation or carotid dissection (Figures 2(c) and 2(d)).

Laboratory analysis showed the patient's hemoglobin 7.6 g/dL, hematocrit 23%, ferritin 4.6 ng/mL, RBCs3.16 × 10^6^/uL,MCV 72.8 fL, and MCH 24.1 pg/cell, which were all below normal limits, and along with the peripheral blood smear, displayed microcytic hypochromic anemia. The patient also had thrombocytosis with a platelet count of 512,000. The patient's lipid panel was normal, consisting of LDL 67 mg/dL and HDL 42 mg/dL. The other laboratory parameters including white blood count, chemistry panel, urea, creatinine, and anion gap were also within normal limits.

**Figure 3:**
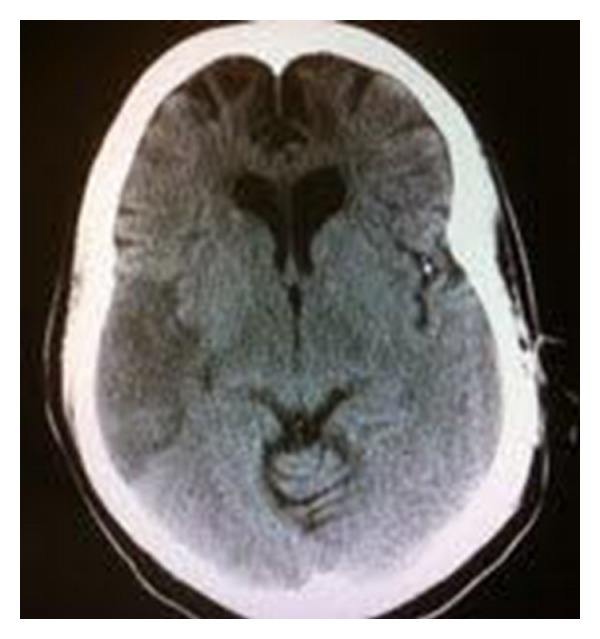
CT scan of the head without contrast showing a larger right MCA infarction compared Figure 1.

During the hospital stay, the patient was found lying on the floor unresponsive, with bowel incontinence. The patient's eyes deviated to the right side, left face weakness, and left upper extremity with 0/5 on MRC (Medical Research Council) scale for motor strength. Two minutes prior to this event, patient was seen ambulating in her room. She gradually became responsive over time and was loaded with Phenytoin for the suspicion of seizure. She was then transferred to the ICU care. A repeat CT scan of the head (Figure 3) showed a large right MCA territory acute nonhemorrhagic infarction with significant increase in size seen on previous MRI. The patient's hemoglobin was 6.9 g/dL during this event. Due to her history of uterine fibroma, the patient underwent a transvaginal ultrasound, which ruled out active bleeding. The patient received two units of packed red blood cells (PRBCs) and her hemoglobin stabilized throughout the remainder of the hospital course between 9-10 g/dL. She was enrolled into physical and speech therapy and was discharged on the fifteenth day with residual left arm upper weakness.

Note, all other possible confounding factors for stroke were excluded by doing additional tests, which were all normal, including hemoglobin electrophoresis, paroxysmal nocturnal hemoglobinuria marker, protein S and C, antithrombin III, factor Leiden V, erythrocyte sedimentation rate, antinuclear antibody, rapid plasma reagin, anticardiolipin antibody, Vitamin B12 levels, homocysteine levels, prothrombin and partial prothrombin times, hemoccult stool, TEE, and lupus anticoagulant antibodies.

## 3. Discussion

Strokes can occur at any age and although the majority occurs over the age of 65, nearly one-fourth of strokes occur in people under the age of 65. Some common causes in this population include cardioembolism, hematologic disorders, substance abuse, trauma, dissections, oral contraceptive use, connective tissue disorders, pregnancy and postpartum states, and migraine. However, the cause of stroke in young patients remains undetermined in about 30% of cases [2]. Therefore, there needs to be a greater focus on discovering the other risk factors such as IDA in this population. IDA has been suggested to have an association with stroke, but few cases have proven it thus far.

IDA has been associated with cerebrovascular disease under three conditions. First, it has been found in patients with venous sinus thrombosis [3] or central retinal vein thrombosis [4], possibly due to secondary thrombocytosis [5]. Second, severe anemia may cause reversible focal deficits on an anemic hypoxic basis [6], sometimes in the setting of severe atherosclerotic disease. Third, IDA has been associated with cerebral infarctions, presumably on an arterial basis [7, 8] either with the evidence of thrombus formation or hypoxic basis.

Childhood ischemic strokes associated with IDA have been studied in greater depth than IDA-associated strokes in young adults. Three physiological mechanisms explaining IDA to childhood ischemic stroke include a hypercoagulable state secondary to IDA, thrombocytosis secondary to IDA, and anemic hypoxia induced by IDA [9]. Hartfield et al. reported a series of six children, between 6–18 months of age with an ischemic stroke or venous thrombosis after a viral prodrome. All patients had iron deficiency as a consistent finding among the group, and other known etiologies of childhood stroke were excluded [10]. These risk factors could possibly be a cause of stroke in the adult population as well.

Cases of carotid thrombus associated with iron deficiency anemia and thrombocytosis have been reported in adults. One reason is due to an anemic patient needing more blood flow to the brain to compensate for the lack of oxygen. Therefore, the increase in blood flow can cause endothelial vessel damage, causing a cascade of thrombus formation to occur. Akins et al. reported three case reports in which young women with severe IDA and thrombocytosis secondary to menorrhagia develop carotid artery thrombosis. The patient's profile and workup were similar to our case. The three women in these cases were 44, 20, and 39 years old, and the hemoglobin levels ranged between 6.3–7.1 g/dL [1]. Each of the case reports had evidence of thrombus by angiography due to suspicion of thrombus-induced stroke. They also ruled out other common causes for stroke by performing similar tests such as in our case.

Our paper shows an example of a hypoxia-induced stroke secondary to IDA in a young woman with menorrhagia. Thrombus formation was ruled out as the MRA showed no evidence. As all other known causes for stroke were ruled out, the patient's IDA is a reasonable cause for her stroke. Iron deficiency decreases the amount of hemoglobin, which consequently decreases the amount of oxygen in the blood resulting in low oxygen delivery to the brain. This causes hypoxic conditions in the brain, leading to death of brain tissue. Thus, we suggest a possible relationship between IDA and ischemic stroke in young adults.

Our patient had presented to the emergency department with hemoglobin of 7.6 g/dL, which was not focused on as a possible cause for stroke at that time. The patient then became unresponsive with sudden onset of left extremity weakness during her continued hospital stay with a lower hemoglobin of 6.9 g/dL. The patient was transfused with two units of PRBCs and thereafter she gradually showed improvement in motor strength and speech. After ruling out the common causes of stroke, it became clear that the low hemoglobin was most likely the culprit. It is likely that the persistent low hemoglobin and upright position of the patient may have caused hypoxia and an acute ischemic attack in her brain due to lack of oxygen delivery. This is an example of the hypoxia-induced mechanism, which was supported by the development of larger infarction in the right MCA region.

Anemia is often overlooked as an important factor in the setting of ischemic stroke, which upon correction may significantly improve the outcome. Huang et al. focused on the influence of anemia on clinical presentation and outcome of patients with first-ever atherosclerosis-related ischemic stroke. It has been shown that within three years of initial onset of first-ever atherosclerosis-related ischemic stroke, the mortality rate was significantly higher in patients who had anemia at the time of admission [11]. This topic clearly needs serious attention and further research.

If our patient had been treated for anemia on admission, this may have prevented her from progressing to a larger stroke, thus providing a more favorable outcome. Therefore, considering such risk factors like anemia is important in the prevention of such devastating and debilitating events.

## 4. Conclusion

This case has been reported because of the unique sequence of events that led to an interesting correlation between IDA and hypoxia-induced ischemic stroke. When stroke occurs in younger individuals, we need to search for other risk factors, such as dissection, AV malformations, coagulopathies, connective tissue disorders, OCP use, and now discovering that IDA should not be overlooked. IDA is a common concern predominantly in younger women, which must be addressed as a risk factor for stroke. In this case, transfusing the patient and thereby curing the anemia is what most likely saved the patient's life and prevented her from further possible ischemic strokes. If anemia was a known risk factor and our patient's anemia was corrected upon admission, the outcome may have been favorable, preventing the residual left extremity weakness. Therefore, a standard set of guidelines should be made regarding transfusions in anemic patients in the setting of stroke. IDA has been overlooked as a risk factor for ischemic stroke and needs to be further studied with greater focus.

## Abbreviations

CT: Computerized tomography
MRI: Magnetic resonance imaging
MRA: Magnetic resonance angiogram
IDA: Iron deficiency anemia
RBC: Red blood cell
MCA: Middle cerebral artery
MCV: Mean corpuscular volume
MCH: Mean corpuscular hemoglobin
HDL: High-density lipoprotein
LDL: Low-density lipoprotein
MRC: Medical research council
TEE: Transesophageal echocardiography
ICU: Intensive care unit
PRBC: Packed red blood cell
OCP: Oral contraceptive pill
AV: Arterio venous.

## References

[1] Akins PT, Glenn S, Nemeth PM, Derdeyn CP (1996). Carotid artery thrombus associated with severe iron-deficiency anemia and thrombocytosis. *Stroke*.

[2] Marcoux M (2000). Stroke in young adults. *Colorado Neurological Institute Medical Journal*.

[3] Aoki N, Sakai T (1989). Cerebral sinus thrombosis in patients with severe iron deficiency anaemia due to myoma uteri. *Acta Neurochirurgica*.

[4] Shibuya Y, Hayasaka S (1995). Central retinal vein occlusion in a patient with anorexia nervosa. *American Journal of Ophthalmology*.

[5] Schloesser LL, Kipp MA, Wenzel FJ (1965). Thrombocytosis in iron-deficiency anemia. *The Journal of Laboratory and Clinical Medicine*.

[6] Young RSK, Rannels DE, Hilmo A (1983). Severe anemia in childhood presenting as transient ischemic attacks. *Stroke*.

[7] Knizley H, Noyes WD (1972). Iron deficiency anemia, papilledema, thrombocytosis, and transient hemiparesis.. *Archives of Internal Medicine*.

[8] Alexander MB (1983). Iron deficiency anemia, thrombocytosis, and cerebrovascular accident. *Southern Medical Journal*.

[9] Maguire JL, Deveber G, Parkin PC (2007). Association between iron-deficiency anemia and stroke in young children. *Pediatrics*.

[10] Hartfield DS, Lowry NJ, Keene DL, Yager JY (1997). Iron deficiency: a cause of stroke in infants and children. *Pediatric Neurology*.

[11] Huang WY, Chen IC, Meng L, Weng WC, Peng TI (2009). The influence of anemia on clinical presentation and outcome of patients with first-ever atherosclerosis-related ischemic stroke. *Journal of Clinical Neuroscience*.

